# Effects of Ingesting Food Containing Heat-Killed *Lactococcus lactis* Strain Plasma on Fatigue and Immune-Related Indices after High Training Load: A Randomized, Double-Blind, Placebo-Controlled, and Parallel-Group Study

**DOI:** 10.3390/nu15071754

**Published:** 2023-04-04

**Authors:** Yuta Komano, Kosuke Fukao, Kazunori Shimada, Hisashi Naito, Yoshihiko Ishihara, Toshio Fujii, Takeshi Kokubo, Hiroyuki Daida

**Affiliations:** 1 Kirin Holdings Company, Limited, Tokyo 164-0001, Japan; 2 Graduate School of Health and Sports Science, Juntendo University, Chiba 270-1695, Japan; 3 Department of Cardiovascular Biology and Medicine, Juntendo University Graduate School of Medicine, Tokyo 113-8421, Japan; 4 School of Science and Technology for Future Life, Department of Humanities and Social Sciences, Tokyo Denki University, Tokyo 120-8551, Japan; 5 Faculty of Health Science, Juntendo University, Tokyo 113-8421, Japan

**Keywords:** *Lactococcus lactis* strain Plasma, fatigue, dendritic cell, high training load

## Abstract

*Lactococcus lactis* strain Plasma (LC-Plasma) is a unique lactic acid bacterium that activates plasmacytoid dendritic cells (pDCs). We evaluated the effect of LC-Plasma on fatigue indices and dendritic cells activity in athletes after 14 days’ continuous exercise load. Thirty-seven participants were divided into two groups and consumed placebo (PL) or LC-Plasma capsules (containing 100 billion cells) daily for 14 days. Maturation markers on dendritic cells, blood parameters, physiological indices, and fatigue-related indices were recorded on days 1 and 15 (before and after exercise). Cumulative days of symptoms relating to physical conditions were also recorded during the continuous exercise period. We observed that CD86 as a maturation marker on pDCs was significantly higher and that cumulative days of fatigue were significantly fewer in the LC-Plasma group than in the Placebo group on day 15. We also conducted 2 h ergometer exercise on day 15 to evaluate fatigue. The results showed that autonomic fatigue parameters (LF/HF) were significantly lower in the LC-Plasma group. These results suggest that LC-Plasma supplementation alleviates fatigue accumulation and increases pDC activity caused by a continuous high training load.

## 1. Introduction

Low-to-moderate-intensity exercise is effective for the maintenance and promotion of health [1,2]. However, the high-intensity or strenuous exercise that elite athletes routinely undergo during training may reduce quality of life and may cause sleep disturbance, depression, and eating disorders [3]. Upper respiratory tract infection (URTI) accounts for approximately 65% of non-traumatic symptoms in athletes who routinely perform strenuous exercise [1,4] and is considered to be caused by reduced immunity after exercise [5]. Strenuous exercise has been reported to reduce the amount of secretory immunoglobulin (Ig) A that contributes to infection prevention, leading to reduced activities of natural killer cells and neutrophils that are involved in the elimination of bacteria and viruses that invade the body [6,7,8,9,10]. In addition, strenuous exercise causes fatigue. Fatigue is a complex physiological and pathological phenomenon associated with feelings of exhaustion or tiredness and declining physical performance [11], and is categorized as peripheral and central fatigue [12]. Although the direct causal relationship between immunity and fatigue is not clear, both are characteristic physical changes that occur after exercise. As such, reduced immunity and fatigue are serious health concerns in athletes who frequently perform high-intensity exercise, and effective prevention methods that can be easily and routinely implemented are desired.

Lactic acid bacteria have been attracting interest as food components that can solve health problems in athletes, such as reduced immunity and fatigue. Various strains of Lactic acid bacteria are used in yogurt, cheese, and pickles. It has been shown that lactic acid bacteria are extremely safe through meal experiences and they are used in many health foods. For instance, *Lactobacillus* intake led to a reduced prevalence of URTI, maintenance of exercise performance, and reduced fatigue in clinical studies with athletes [13,14].

*Lactococcus lactis* strain Plasma (LC-Plasma) is a synonym of *Lactococcus lactis* subsp. *lactis* JCM 5805. It is a lactic acid bacterium that has been reported to activate plasmacytoid dendritic cells (pDCs) by stimulating toll-like receptor 9 (TLR-9) signaling [15]. pDCs do not react to common lactic acid bacteria, but LC-Plasma is characteristically phagocytosed by pDCs. The phagocytosed LC-Plasma, with DNA as an active component, stimulates TLR-9, thereby activating pDCs. pDCs play an important role in immune function, especially for virus infection, by the production of type I and III interferons [16]. As DNA is an active component, this effect of LC-Plasma is exhibited equally in both live and heat-killed states [15]. A clinical study in healthy participants reported that live or heat-killed LC-Plasma intake improved the activity of pDCs and also improved work performance [17,18]. In addition, in vivo experiments confirmed that the continuous intake of LC-Plasma improved spontaneous motor activity after exercise, and suppressed an increase in fatigue and the muscle decomposition index [19]. Based on these findings, we considered that LC-Plasma could be a useful lactic acid bacterial material that could exhibit positive effects on health challenges in athletes.

In our previous study, we evaluated the effect of heat-killed LC-Plasma supplementation on continuous exercise. The participants, who belonged to three types of university sports clubs, ingested placebo (PL) or LC-Plasma for 2 weeks and performed strenuous exercise during the test period. As a result, the LC-Plasma group showed reduced cold-like symptoms, enhanced pDC activity, and reduced subjective fatigue accumulation after continuous exercise [20]. However, there were some limitations, which may have affected the results of the study. Firstly, the content of the continuous exercise varied between the sports clubs. Secondly, the effect on fatigue was observed only in a subjective results index generated using a diary questionnaire.

In this study, we conducted a randomized, double-blind, placebo-controlled study with 14 days of continuous exercise to confirm the reproducibility of the effects of LC-Plasma on pDC activity and fatigue. To address the limitations of our previous study, all the participants belonged to a single sports club and received the same type of training. In addition, the participants underwent an additional strenuous single exercise session to evaluate fatigue on day 15. In addition, both subjective and objective analysis were employed.

## 2. Materials and Methods

### 2.1. Research Ethics and Participants

The present study adhered to the principles of the Declaration of Helsinki and the Ethical Guidelines for Medical and Health Research Involving Human Subjects. Documents such as the study protocol, the investigator’s brochure, the explanation form, and the consent form were submitted to the ethics committee of the Faculty of Health and Sports Science, Juntendo University, and the study was conducted following approval by the committee (approval number: 29–13). This study was registered at the University Hospital Medical Information Network Clinical Trial Registry as UMIN000028717.

The study design was a randomized, placebo-controlled, double-blinded, parallel-group trial for healthy Japanese student volunteers. In August 2017, we recruited students from Juntendo University, who were aged 18 years or older, who belonged to the long-distance track and field team, and who regularly exercised at least four times a week, by posting a notice at the Faculty of Health and Sports Science, Juntendo University. The experiment was conducted from late August to September 2017.

The minimum sample size was estimated on the basis of CD86 expression, which is one of the activation markers of pDCs. Our previous study reported that the standard deviation for CD86 was 20%, and the difference between the pre- and post- ingestion mean value was 15% [17]. According to these parameters, we calculated that a sample size of 16 in each arm would be required to achieve at least 80% power (β ≥ 0.8) with statistical significance (α ≤ 0.05) in a paired *t*-test. On the assumption that a few participants might withdraw from the study after enrollment, we determined a target number of 20 participants per group (40 in total) to detect the effect on pDC activity. The exclusion criteria were as follows: individuals with serious illnesses (diabetes, kidney/liver disease, heart disease, thyroid disease, adrenal disease, or other metabolic diseases undergoing treatment); individuals who routinely took medications for chronic diseases, individuals who were unable to stop the intake of food containing lactic acid bacteria, bifidobacteria, oligosaccharides, and live bacteria during the study period; and individuals who were considered unsuitable for the study for other reasons by the investigator or the research sponsor. There were 37 male volunteer applicants and no female applicants. We held an explanation meeting for them and obtained informed consent from all the participants. Based on the preliminary test results, 37 men were determined to be eligible. To avoid bias related to age, body height, weight, body mass index (BMI), and pDC activity at the time of the preliminary test, the person responsible for statistical analysis assigned the participants into two groups by computerized randomization using the stratified permuted block randomization method. The participants were assigned to the control food intake group (PL group, n = 19) or the investigational food intake group (LC-Plasma group, n = 18). The allocation table was sealed by the controller and securely stored until the cases were fixed and analytical results were summarized.

### 2.2. Test Food

The study used LC-Plasma capsules containing heat-killed dry LC-Plasma (Kyowa Hakko Bio CO., Ltd., Tokyo, Japan) (≥1.0 × 10^11^) and cornstarch (Showa Sangyo Co., Ltd., Tokyo, Japan) in No. 3 size capsule, and PL capsules containing only cornstarch. The LC-Plasma was produced by culturing in a medium, separation, washing, pasteurization, and drying. These capsules were prepared so that the investigators and participants could not differentiate between the LC-Plasma and the PL by appearance or smell.

### 2.3. Study Design

**Figure 1:**
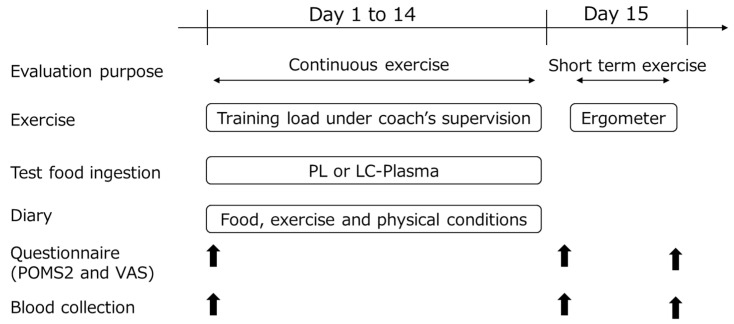
Experimental design. Arrows indicate points when questionnaires and blood collections were taken.

The schematic representation of the study is shown in Figure 1. The participants performed daily continuous exercise from day 1 to 14, followed by the short-term 2 h exercise on day 15. The test foods were ingested for 14 days. The participants recorded diaries during the continuous exercise period. Hematological and physiological indices were measured, and the questionnaire was conducted on days 1 and 15 (before and after exercise). The period from day 1 to 15 before exercise was set to evaluate the effects on continuous exercise, and day 15 was set to evaluate the effects of short-term exercise.

The participants were instructed to maintain their daily habits (e.g., for diet and sleep) and to avoid foods containing a large amount of lactic acid bacteria (e.g., yogurt, fermented cheese, pickles, and lactic acid bacteria supplement) within the test period. For three days prior to the study, participants stayed at rest without exercise to unify their initial state as much as possible. During the 14 days of ingesting the test foods, the participants performed exercise under the supervision of a track and field coach every day. The experimental period was same as the previous study [20], and it was confirmed that DC activity decreased significantly after 14 days of continuous exercise in the sports club students, as found in our preliminary study (unpublished data). On day 15, they performed a single 2 h exercise using a cycle ergometer, and exercised in the range of 70–80% of maximum heart rate intensity, which is commonly classified as a high-intensity exercise level [21,22,23,24].

In the participants’ diary, they recorded test food intake, exercise (type and duration), meal details, and their physical conditions. From the exercise record, the amount of exercise was evaluated as metabolic rate–hour (MET-h), an index of physical activities based on energy expenditure [25]. The MET-h was calculated by selecting METs from the standard METs table for each exercise and multiplying them by the exercise time [26,27].

The blood collection on days 1 and 15 before exercise were conducted at the same time of day (between 08:00 and 10:00 a.m.). The blood samples were collected using a vacutainer blood collection tube with a syringe.

### 2.4. Evaluation of pDC and mDC Activity

Peripheral blood mononuclear cells (PBMCs) were isolated from the blood [17]. To evaluate pDC activity, the PBMCs were incubated with anti-human CD123-FITC (AC145) (Miltenyi Biotec., Bergisch Gladbach, Germany), BDCA4-APC (AD-17F6) (Miltenyi Biotec.), CD86-PE (IT2.2) (eBioscience, San Diego, CA, USA), and HLA-DR-PerCP (L243) (BD Bioscience, Franklin Lakes, NJ, USA) and with anti-human Lineage Cocktail1-FITC (Lin1) (CD3, CD14, CD16, CD19, CD20, CD56) (MφP9, NCAM16.2, 3G8, L27, SJ25C1, SK7) (BD Bioscience), CD11c-APC (MJ4-27G12) (Miltenyi Biotec.), CD1c-PE-Cy7 (L161) (BioLegend, San Diego, CA, USA), CD86-PE (IT2.2) (eBioscience), and HLA-DR-PerCP (L243) (BD Bioscience) to evaluate mDC activity. CD123 positive + BDCA4 positive cells were defined as pDCs, and Lin1 negative + CD11c positive + CD1c positive cells were defined as mDCs. The expression intensities of the cell surface molecules CD86 and HLA-DR were used as a marker of pDC and mDC activation. Data were collected by flow cytometer (FACS Cant II, BD Biosciences), and FlowJo software (Treestar, Ashland, OR, USA) was used for data analysis.

### 2.5. Evaluation of Hematological Indices

To investigate the influence of exercise load and LC-Plasma intake on the objective parameters, hematological indices known to be associated with exercise-induced inflammation and oxidative stress were measured. The levels of transforming growth factor-β (TGF-β) and interleukin-6 (IL-6) were measured using an ELISA kit (R&D systems). The levels of cathepsin L, adrenaline, hydroxy-8-deoxyguanosine (8-OHdG), testosterone, and leptin were measured using separate ELISA kits (R&D systems, Minneapolis, MN, USA; R&D systems; Arigo Biolaboratories Corp, Hsinchu City, Taiwan; Northwest Life Science Specialties, Portland, OR, USA; and R&D systems, Cosmo Bio, Tokyo, Japan, respectively). The levels of creatine phosphokinase (CPK) were measured by Hoken Kagaku, Inc. (Kanagawa, Japan) using the Japan Society of Clinical Chemistry standardization method.

### 2.6. Evaluation of Physiological Indices

The low frequency to high frequency ratio (LF/HF), which is indicative of sympathetic to parasympathetic autonomic balance, was measured to assess the degree of fatigue, using the autonomic measurement device VM302 (Hitachi Systems, Tokyo, Japan) [28]. Participants insert both index fingers into holes in the device and remain at rest for few minutes. The pulse waves and electrocardiography are measured from finger blood flow, heart rate variability is analyzed from the results, and autonomic nerve function is measured as LF/HF. The power spectral density obtained by use of the maximum entropy method was divided into the LF component, within the range of 0.04–0.15 Hz, and the HF component, within the range of 0.15–0.4 Hz. The mean values of HR, LF, HF, and LF/HF obtained in 30 s were calculated.

### 2.7. Participant Diary and Questionnaire

Participants were asked to record the degree of each clinical symptom (physical conditions, nasal congestion/nasal discharge, pharyngeal pain, cough, joint pain, chill, fatigue, malaise, and muscle pain) daily using a five-point Likert scale modified with reference to a previous report [29]. Participants recorded a self-assessment on the severity of each symptom using a five-degree scale of severity (1: very severe; 2: severe; 3: moderate; 4: mild; 5: none). We compared the cumulative incidence days of all the participants by separating the results based on two categories of severity: with symptoms (scale 1 to 3) and without symptoms (scale 4 and 5) to test whether intake of LC-Plasma affected the duration of severe symptom onset.

To evaluate the participants’ mood during the intake period, the Profile of Mood States 2 (POMS2) test [30] was used. The POMS2 test comprises 35 questions and uses a five-point Likert scale to assess mood state. The test has the following subscales: anger–hostility, confusion–bewilderment, depression–dejection, fatigue-inertia, tension–anxiety, vigor–activity, and friendliness. Total mood disturbance (TMD) was also calculated, which is a summary measure of the subscales other than friendliness. Except for vigor–activity and friendliness, a low POMS2 score indicates a better state of mood.

The visual analog scale (VAS) questionnaire was used to evaluate subjective conditions. The participants were asked about fatigue or physical state questions. In each of the questions, subjects were instructed to mark a spot on a 100 mm straight line according to their feelings (left end 0 = best, right end 1000 = worst). The distance of the mark from the left end was measured and used as the VAS score. A low VAS score indicates a good mood state.

### 2.8. Statistical Analysis

The primary endpoints were pDC activity, mDC activity, participant diary records, POMS2, and TGF-β, IL-6, CPK, cathepsin L, and adrenaline levels between the PL group and the LC-Plasma group on day 15. The secondary endpoints were VAS score, LF/HF, and 8-OHdG, testosterone and leptin levels. Data were expressed as the mean and standard deviation for each intake group. For intragroup comparisons of hematological and physiological indices, the paired *t-*test was used. For intergroup comparisons, Student’s *t-*test was used. For intragroup comparisons based on the questionnaire, Wilcoxon’s test was used. For intergroup comparisons, the Mann–Whitney U test was used. For intragroup comparisons of the cumulative number of days of physical conditions and fatigue indices in the participants’ diary, the Chi-squared test was used. For all the statistical analyses, the Excel Toukei software program (Social Survey Research Information, Tokyo, Japan) was used. For all the tests, a *p* value of <0.05 was considered statistically significant.

## 3. Results

### 3.1. Participants’ Characteristics

**Figure 2:**
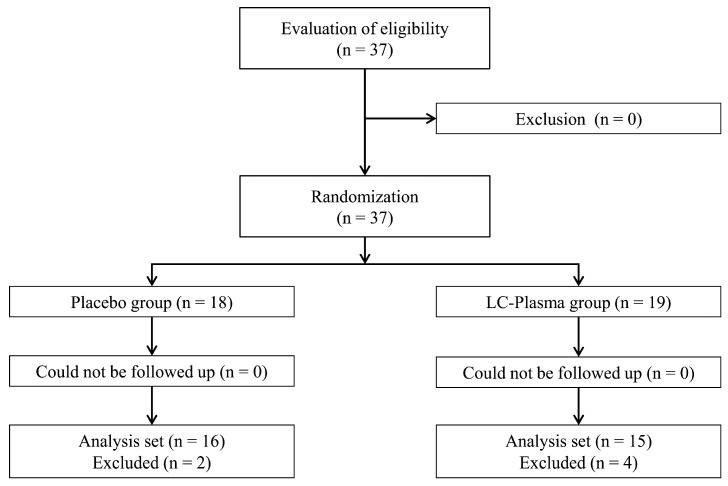
Workflow from participant enrolment to analysis.

Figure 2 shows the workflow of the study from participant enrolment to analysis. Table 1 presents the background information of the participants. During the intake period, no participant experienced adverse event or asked to discontinue the study. However, the participant diary confirmed that four participants did not ingest the test food for 3 or more consecutive days and two participants did not record their level of exercise for 7 or more days. These participants were excluded from the assessment, as the principal investigator considered this appropriate to secure the reliability of the data. The other 31 participants (16 in the PL group and 15 in the LC-Plasma group) were included in the analysis. There were no significant intergroup differences in participants’ age, body height, weight, or BMI (Table 1). The average amount of exercise per week for the intervention period (PL group: 89.9 ± 17.7 MET-h/week; LC-Plasma group: 94.5 ± 21.5 MET-h/week) and average heart rate based on use of a heart rate monitor (Polar Electro, Tokyo, Japan) during the single exercise session on day 15 (PL group: 144.7 ± 11.5 bpm; LC-Plasma group: 141.2 ± 9.3 bpm) were not significantly different between the groups. The majority of exercise during the period was long-distance running. There was no adverse event associated with the test food. Therefore, there was no marked difference in participant background between the groups and the study was appropriately conducted. The following analyses were performed on the 31 participants.

### 3.2. Assessment for Continuous Exercise

We first analyzed the effect of LC-Plasma on continuous exercise. There was a significant decrease in pDC activity (CD86) on day 15 compared with that on day 1 in both groups, and on day 15, the level in the LC-Plasma group was significantly higher than that in the PL group (Table 2). There was also a significant decrease in pDC activity (HLA-DR) on day 15 compared with that on day 1 in the PL group only. There was no significant difference in mDC activity for either CD86 or HLA-DR.

The cumulative days of fatigue in the participants’ diary revealed a significant decrease in the LC-Plasma group compared with that in the PL group. The cumulative days of joint pain showed a significantly higher in the LC-Plasma group compared with that in the PL group (Table 3). The score of the Friendliness item in POMS2 significantly decreased in the LC-Plasma group only (Table 4). No significant differences in VAS were found in the intra- and intergroup comparisons.

Hematological and physiological indices, commonly known to be associated with fatigue, including TGF-β, CPK, adrenaline, and 8-OHdG levels, and LF/HF showed significant changes within each group; however, no significant difference were found in the intergroup comparison (Table 5).

### 3.3. Assessment for Strenuous Single Exercise

Next, we analyzed the effect of LC on a single exercise session following long-term exercise. There were no significant differences in DC activity and the POMS2 score in the intra- or intergroup comparisons. The score for the item “Are you tired?” and “Are you feeling physical listlessness?” in VAS showed a significant increase in the intragroup comparison in both groups, and “Are you feeling muscle pain?” showed a significant increase in the intragroup comparison only in the PL group (Table 6). The hematological and physiological indices, IL-6, adrenaline, and leptin level in the blood were significantly different in the intragroup comparison in both groups (Table 7). The testosterone level was significantly decreased in the PL group only. Furthermore, LF/HF significantly decreased in the LC-Plasma group compared with those in the PL group.

## 4. Discussion

The primary aim of this study was to confirm the effects of LC-Plasma on fatigue caused by a continuous high training load. All the participants in this study belonged to the same sports club. Since the participants had had 3 days of rest without exercise before the start of the test, it is considered that the fatigue state at the start of the experiment was stable. During the intake period of continuous exercise, the average intensity of exercise of the 31 participants was 13.2 ± 2.8 MET-h/day. This is higher than the intensity in our previous study (12.4 ± 3.9 MET-h/day). It has been reported that individuals who run more than 14 km per day (approximately 14 MET-h/day) are more susceptible to URTI [31]. The intensity of this study was close to this value. A significant increase in CPK, a typical blood index known to rise after strenuous exercise with muscle damage [32], was observed after the continuous exercise. From this result, we considered that the design of this continuous exercise was sufficient to study the effect of LC-Plasma on fatigue.

Our results suggested that the CD86 of pDCs in the LC-Plasma group was significantly higher than the level found in the PL group after continuous exercise. The HLA-DR in pDCs significantly decreased in the PL group only in the intragroup comparison. As for the sense of fatigue, cumulative days of fatigue in the participant diary significantly decreased in the LC-Plasma group. The scores of Fatigue–Inertia in POMS2 and “Are you tired?” in VAS showed a worsening trend in the PL group only. These results are similar to those of our previous study [20]. There are many reports on the link between immunity and fatigue [33,34,35,36]. Therefore, LC-Plasma intake increased pDC activity and maintained immunity, which may have resulted in the improvement in the subjective indices of fatigue. The relationship between exercise and DCs has been reported in several studies. Esquius et al. reported that acute aerobic exercise increased the ratio of mDCs to pDCs, and that extra-virgin olive oil intake suppressed this effect [37]. On the other hand, there are no reports that evaluate the pDC activity state by exercise or the effect of food material intake on them. Therefore, this was considered to be a characteristic effect of LC-Plasma.

We also assessed the effects of LC-Plasma intake on the objective indices (TGF-β, IL-6, cathepsin L, adrenaline, 8-OHdG, testosterone, leptin, and CPK) associated with fatigue. Some factors in the hematological indices were significantly changed in the intragroup comparison after both the continuous and single exercise; however, no significant difference was observed between the groups. Adrenaline was the index that changed after both the continuous and single exercise in the intragroup comparison. Adrenaline is released with exercise and helps the body to resist stress conditions. It affects the cardiovascular system, producing a rapid powerful vasopressor effect, and increasing heart rate, the force of contraction, the respiratory rate, and blood flow towards the brain, heart and skeletal muscle [38]. It is known that adrenaline secretion correlates with exercise intensity [39,40]. The single exercise session in this study was high-intensity, requiring 70–80% of maximum heart rate, and it was considered that adrenaline increased, reflecting this level of exercise intensity. In the evaluation of clinical symptoms associated with URTI, LC-Plasma intake did not improve the cumulative days of nasal congestion/nasal discharge the prolonged exercise, contrary to our previous study. This result is considered to be due to the inability to assess the seasonal effect of LC-Plasma on URTI. In the previous study, which was conducted in winter, 15.7% of the days had symptomatic nasal disorder factors, compared with only 1.4% in the present study, which was conducted in summer. The low risk of cold-like illness in summer made it difficult to detect the effect of LC-Plasma. Considering the fact that the effect of LC-Plasma on the fatigue factor was similarly confirmed in both studies, we consider that the records of the participants in the questionnaire were conducted appropriately.

We also performed a short-term single exercise test after the continuous exercise for the purpose of assessing the impact of LC-Plasma on fatigue in more detail. However, no significant difference in CD86, HLA-DR, or subjective analysis of fatigue was observed in the intergroup comparison. This may be due to the insufficient intensity of the single exercise session, because no change in CPK was observed. However, it should be noted that in the index of the sympathetic and parasympathetic autonomic balance, the LF/HF value was significantly low and improved in the LC-Plasma group after the single exercise session, although no significant difference was detected between the groups after the continuous exercise. LF/HF has been reported to be linked with fatigue [41,42,43]. Since the autonomic nervous system fluctuates over a short time, LF/HF is often used as a short-term fatigue index of exercise [44,45]. Similarly to the results of this study, it has been reported that the increase in LF/HF after exercise is suppressed by 400 mg of caffeine intake [46]. It may have been easier to detect the effect of LC-Plasma after the single exercise session. In addition, testosterone, which has been reported to be linked to fatigue and vitality [47], significantly decreased in the Placebo group only, exacerbating fatigue. The autonomic nervous system has been reported to be linked to activation of the immune signal TLR9. When administered with a sympathetic nerve-activating agent, atropine, TLR9 knockout mice showed a sympathetic dominant phenotype, such as an increased pulse rate, more than was the case in wild-type mice [48]. Thus, it has been suggested that TLR9 activates the parasympathetic nervous system. The main mechanism of action of LC-Plasma has been reported to activate TLR9 [15]; therefore, it was considered that LC-Plasma may activate the parasympathetic nervous system by stimulating TLR9, which resulted in reducing LF/HF. In order to elucidate the relationship between LC-Plasma administration and the autonomic nervous system, more precise mechanism investigation, such as physiological function evaluation using TLR9 knockout mice, are required in the future.

We set the target sample size at 20 subjects in each group, when the power was set at 0.8 based on the results of CD86 expression of previous experiments. As a result of the final sample size of each group being 15 and 16 due to the exclusion of participants, the standard deviation was larger than in past measurements. Therefore, the power of this experiment was 0.6, which was lower than expected. A larger number of subjects may have been required for a more appropriate LC-Plasma evaluation.

There are some limitations to this study that may affect the results. The subjects were limited to athletes aged around 20 years, who performed exercise routinely. The intensity of exercise was also limited. The tracing of lifestyle factors such as meal records, heart rate, and blood pressure during the study period are insufficient to omit the possibility of confounders. In addition, since the short-term single exercise was performed the day after the end of the continuous exercise, it is possible that the influence of the continuous exercise on the subject’s fatigue accumulation cannot be completely excluded.

In the future, further studies are needed with participants from more diverse backgrounds, at a more appropriate timing, with stricter lifestyle controls, and with more hematological investigations to reveal the impact of LC-Plasma on fatigue.

## 5. Conclusions

The present study demonstrated that supplementation with LC-Plasma reduced the accumulation of subjective feelings of fatigue after continuous exercise in participants who routinely exercised, potentially by maintaining pDC activity. In addition, the possibilities of improvements in indices of fatigue, such as the normalization of autonomic balance, was demonstrated in a strenuous single exercise session.

## Figures and Tables

**Table 1:** Participants’ characteristics.

	PL Group	LC-Plasma Group	*p* Value
Age (years)	19.8 ± 1.3	19.9 ± 1.2	0.800
Body height (cm)	171.6 ± 3.7	171.5 ± 5.4	0.935
Weight (kg)	56.3 ± 1.8	56.4 ± 4.1	0.989
BMI ^a^ (kg/m^2^)	19.1 ± 0.9	19.2 ± 1.0	0.972

Values are mean ± SD. ^a^: Body mass index (kg/m^2^) = weight (kg)/body height (m)/body height (m).

**Table 2:** The expression intensities of cell surface molecules CD86 and HLA-DR as the DC activity marker on days 1 and 15.

	Group	Day 1	Day 15	*p* Value (Effect Size)	
				Intragroup Comparison	Intergroup Comparison
pDC CD86	PL	312 ± 132	196 ± 55	0.000 ** (1.185)	0.015 * (0.963)
	LC-Plasma	347 ± 144	271 ± 101	0.047 * (0.633)	
pDC HLA-DR	PL	4918 ± 1265	4246 ± 1328	0.009 ** (0.535)	0.510 (0.246)
	LC-Plasma	4764 ± 732	4519 ± 920	0.189 (0.305)	

Values are mean ± SD. **: *p* < 0.01, *: *p* < 0.05, effect size was presented as Cohen’s D.

**Table 3:** Cumulative days of physical conditions and fatigue symptoms during the continuous exercise period.

Indices	Group	Cumulative Days		*p* Value
		1, 2, 3	4, 5	
Physical conditions ^a^	PL	141	83	0.798
	LC-Plasma	135	73	
Nasal congestion/nasal discharge ^b^	PL	2	222	0.624
	LC-Plasma	4	206	
Pharyngeal pain ^b^	PL	1	223	1.000
	LC-Plasma	1	209	
Cough ^b^	PL	1	223	1.000
	LC-Plasma	0	210	
Joint pain ^b^	PL	1	223	0.034 *
	LC-Plasma	8	202	
Chill ^b^	PL	1	223	0.187
	LC-Plasma	5	204	
Fatigue ^b^	PL	151	72	0.035 *
	LC-Plasma	120	89	
Malaise ^b^	PL	71	153	0.933
	LC-Plasma	68	141	
Muscle pain ^b^	PL	113	111	0.657
	LC-Plasma	100	109	

^a^: Scales were as follows (1: very poor; 2: poor; 3: normal; 4: good; 5: very good). ^b^: Scales were as follows (1: very severe; 2: severe; 3: moderate; 4: mild; 5: none). *: *p* < 0.05.

**Table 4:** Changes in POMS2 scores during the continuous exercise period.

	Group	Day 1	Day 15	*p* Value (Effect Size)	
				Intragroup Comparison	Intergroup Comparison
Anger–Hostility	PL	45.3 ± 4.9	43.2 ± 6.3	0.158 (0.250)	0.708 (0.067)
	LC-Plasma	41.9 ± 3.5	42.6 ± 7.1	0.919 (0.019)	
Confusion–Bewilderment	PL	48.6 ± 5.6	47.2 ± 8.7	0.505 (0.118)	0.519 (0.116)
	LC-Plasma	48.1 ± 9.9	46.5 ± 10.8	0.328 (0.179)	
Depression–Dejection	PL	49.8 ± 4.9	48.3 ± 5.7	0.347 (0.166)	0.826 (0.040)
	LC-Plasma	48.7 ± 3.4	47.9 ± 6.5	0.363 (0.166)	
Fatigue–Inertia	PL	46.8 ± 5.7	50.8 ± 9.5	0.094 (0.296)	0.266 (0.200)
	LC-Plasma	47.2 ± 7.4	47.1 ± 8.6	0.551 (0.109)	
Tension–Anxiety	PL	45.3 ± 8.3	46.7 ± 10.2	0.650 (0.080)	0.193 (0.234)
	LC-Plasma	43.6 ± 8.1	42.3 ± 9.4	0.477 (0.130)	
Vigor–Activity	PL	54.3 ± 9.2	54.6 ± 11.3	0.842 (0.035)	0.751 (0.057)
	LC-Plasma	58.0 ± 9.9	56.4 ± 10.4	0.363 (0.166)	
Friendliness	PL	54.8 ± 10.5	52.6 ± 12.3	0.529 (0.111)	0.842 (0.036)
	LC-Plasma	60.3 ± 6.7	53.9 ± 6.1	0.002 ** (0.559)	
TMD ^a^	PL	10.9 ± 11.8	11.1 ± 15.3	0.605 (0.091)	0.212 (0.224)
	LC-Plasma	6.5 ± 12.5	6.1 ± 18.2	0.414 (0.149)	

Values are mean ± SD. ^a^: Total Mood Disturbance, **: *p* < 0.01, effect size was presented as *r*.

**Table 5:** Changes in hematological and physiological indices during the continuous exercise period.

	Group	Day 1	Day 15	*p* Value (Effect Size)	
				Intragroup Comparison	Intergroup Comparison
TGF-β (pg/mL)	PL	502 ± 95	403 ± 65	0.001 ** (1.256)	0.461 (0.287)
	LC-Plasma	536 ± 127	423 ± 79	0.002 ** (1.106)	
IL-6 (pg/mL)	PL	22.4 ± 17.5	17.0 ± 23.4	0.398 (0.270)	0.291 (0.406)
	LC-Plasma	39.5 ± 34.7	29.1 ± 37.2	0.296 (0.299)	
CPK (U/L)	PL	227 ± 136	307 ± 132	0.012 * (0.617)	0.094 (0.638)
	LC-Plasma	153 ± 74	231 ± 113	0.029 * (0.845)	
Cathepsin L (pg/mL)	PL	1464 ± 1142	883 ± 1074	0.002 ** (0.541)	0.881 (0.058)
	LC-Plasma	1371 ± 1025	943 ± 1077	0.071 (0.421)	
Adrenaline (ng/mL)	PL	0.85 ± 0.77	1.58 ± 1.25	0.021 * (0.726)	0.891 (0.057)
	LC-Plasma	0.82 ± 0.85	1.66 ± 1.66	0.033 * (0.659)	
8-OHdG (ng/mL)	PL	0.46 ± 0.10	0.19 ± 0.08	0.000 ** (3.079)	0.546 (0.291)
	LC-Plasma	0.42 ± 0.07	0.17 ± 0.06	0.000 ** (3.969)	
Testosterone (ng/mL)	PL	23.5 ± 16.0	24.3 ± 15.0	0.451 (0.053)	0.740 (0.128)
	LC-Plasma	28.3 ± 37.2	27.6 ± 35.0	0.625 (0.020)	
Leptin (ng/mL)	PL	50.5 ± 16.2	49.5 ± 19.8	0.793 (0.057)	0.303 (0.395)
	LC-Plasma	54.0 ± 17.6	57.9 ± 24.1	0.355 (0.191)	
LF/HF	PL	1.56 ± 2.83	3.09 ± 4.24	0.014 * (0.670)	0.968 (0.014)
	LC-Plasma	0.54 ± 0.69	3.04 ± 2.79	0.005 ** (1.273)	

Values are mean ± SD. **: *p* < 0.01, *: *p* < 0.05, effect size was presented as Cohen’s D.

**Table 6:** Subjective analysis of fatigue using visual analog scale (VAS) after single exercise.

	Group	Before Exercise	After Exercise	*p* Value (Effect Size)	
				Intragroup Comparison	Intergroup Comparison
Are you tired?	PL	646 ± 187	876 ± 138	0.001 ** (0.603)	0.179 (0.241)
	LC-Plasma	546 ± 185	838 ± 117	0.001 ** (0.623)	
Are you well?	PL	481 ± 227	523 ± 279	0.394 (0.151)	0.406 (0.149)
	LC-Plasma	515 ± 203	598 ± 263	0.140 (0.279)	
Are you feeling physical listlessness?	PL	483 ± 207	696 ± 207	0.007 ** (0.475)	0.874 (0.028)
	LC-Plasma	480 ± 198	722 ± 194	0.009 ** (0.492)	
Are you feeling muscle pain?	PL	364 ± 293	511 ± 226	0.039 * (0.366)	0.737 (0.06)
	LC-Plasma	337 ± 244	464 ± 251	0.064 (0.350)	

Values are mean ± SD. **: *p* < 0.01, *: *p* < 0.05, effect size was presented as *r*.

**Table 7:** Hematological and physiological indices related to fatigue after single exercise.

	Group	Day 15 Pre Exercise	Day 15 Post Exercise	*p* Value (Effect Size)	
				Intragroup Comparison	Intergroup Comparison
TGF-β (pg/mL)	PL	403 ± 65	401 ± 60	0.929 (0.033)	0.230 (0.475)
	LC-Plasma	423 ± 79	438 ± 98	0.495 (0.174)	
IL-6 (pg/mL)	PL	17.0 ± 23.4	76.7 ± 53.2	0.000 ** (1.500)	0.319 (0.376)
	LC-Plasma	29.1 ± 37.2	95.3 ± 48.8	0.000 ** (1.579)	
CPK (U/L)	PL	307 ± 132	311 ± 140	0.680 (0.030)	0.111 (0.605)
	LC-Plasma	231 ± 113	239 ± 102	0.445 (0.077)	
Cathepsin L (pg/mL)	PL	883 ± 1074	575 ± 1205	0.334 (0.279)	0.295 (0.403)
	LC-Plasma	943 ± 1077	1041 ± 1185	0.701 (0.090)	
Adrenaline (ng/mL)	PL	1.58 ± 1.25	4.31 ± 2.68	0.001 ** (1.348)	0.462 (0.274)
	LC-Plasma	1.66 ± 1.66	3.66 ± 2.19	0.000 ** (1.065)	
8-OHdG (ng/mL)	PL	0.19 ± 0.08	0.18 ± 0.11	0.882 (0.107)	0.714 (0.214)
	LC-Plasma	0.17 ± 0.06	0.20 ± 0.08	0.117 (0.439)	
Testosterone (ng/mL)	PL	24.3 ± 15.0	21.4 ± 18.8	0.030 * (0.176)	0.542 (0.234)
	LC-Plasma	27.6 ± 35.0	28.2 ± 38.7	0.817 (0.017)	
Leptin (ng/mL)	PL	49.5 ± 19.8	58.1 ± 20.5	0.011 * (0.441)	0.560 (0.218)
	LC-Plasma	57.9 ± 24.1	63.4 ± 29.2	0.024 * (0.213)	
LF/HF	PL	3.09 ± 4.24	4.23 ± 3.54	0.128 (0.087)	0.009 ** (1.047)
	LC-Plasma	3.04 ± 2.79	1.50 ± 1.28	0.061 (0.734)	

Values are mean ± SD. **: *p* < 0.01, *: *p* < 0.05, effect size was presented as Cohen’s D.

## Data Availability

Not applicable.
